# Supplementary material for: FUS and TAF15 safeguard the critical functions of the ribonucleoprotein network formed by EWSR1 and newly synthesized RNA
Source: bioRxiv. 2026 Mar 26:2026.03.24.713985. Preprint. [Version 1] doi: 10.64898/2026.03.24.713985 (PMC13119339; doi:10.64898/2026.03.24.713985)
Supplement: Supplement 1 [file media-1.pdf]

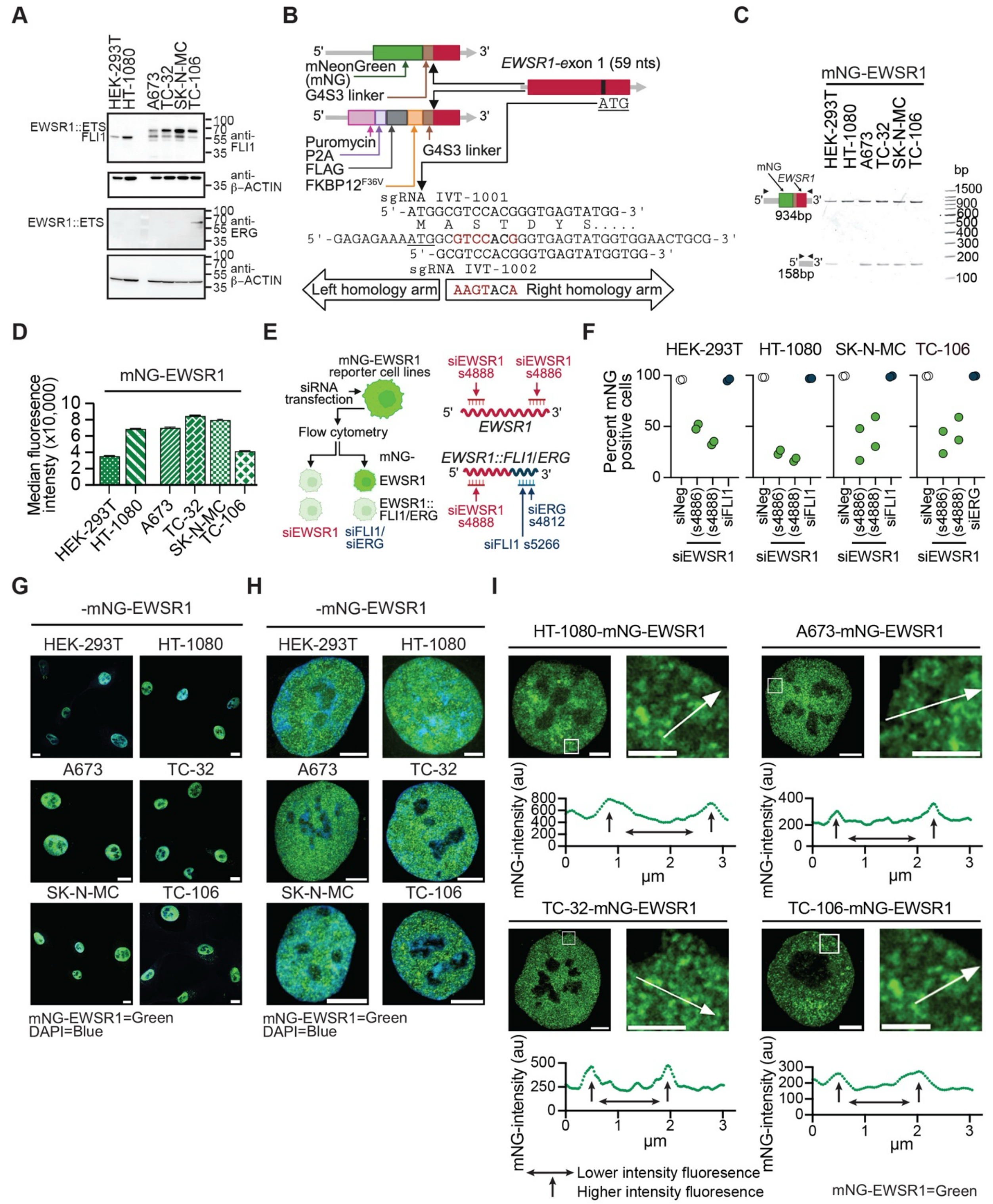

Figure S1: Generation and validation of mNeonGreen-EWSR1 (mNG-EWSR1) reporter cell lines

(**A**) Immunoblot analysis of whole cell lysates from the indicated cell lines probed with antibodies against the indicated proteins.

(**B**) Schematic of the CRISPR-Cas9-mediated modification of the endogenous *EWSR1* locus illustrating insertion of indicated DNA cassettes immediately 5' of *EWSR1* exon 1. Red text denotes sequence changes introduced into exon 1 by donor construct.

(**C**) PCR-based analysis confirming integration of the mNG reporter cassette into the *EWSR1* locus. PCR primers amplify across the donor cassette and flanking 5' and 3' homology arms, indicating. monoallelic modification in HEK-293T cells, biallelic modification in HT-1080 cells, and monoallelic modification in EWS cell lines.

(**D**) Flow cytometry analysis showing median mNG fluorescence intensity in the indicated mNG-EWSR1 reporter cell lines.

(**E**) Schematic of the siRNA-based assay used to determine which *EWSR1* alleles are modified, using siRNAs targeting *EWSR1* or *EWSR1::FLI1/ERG* transcripts[1-3].

(**F**) Flow cytometry analysis following siRNA transfection (two independent replicates). In SK-N-MC and TC-106 cells, reduction in mNG positive cells following transfection of siEWSR1 but not siFLI1 or siERG indicates modification of the unarranged *EWSR1* allele. Corresponding analyses in A673-mNG-EWSR1 and TC-32-mNG-EWSR1 reporter cell lines are reported in Rajan *et al.*, 2024[3].

(**G**) Confocal images of nuclei showing mNG–EWSR1 fluorescence (green) and DAPI-stained DNA (blue). Scale bar, 25 μm.

(**H**) SoRa super resolution confocal images of whole showing mNG-EWSR1 fluorescence (green). Scale bar, 4 μm.

(**I**) SoRa super resolution confocal images showing nuclei (left) and expanded nuclear regions (right) with corresponding line plots. mNG-EWSR1 fluorescence (green) and line plots correspond to the white lines shown in the expanded regions (arrow direction indicates the x-axis orientation of each plot). Horizontal arrowed lines indicate lower intensity mNG-EWSR1 signal. Vertical arrowed lines indicate higher intensity signal. Scale bars, nucleus, 4 μm, expanded region, 2 μm.

(**G-I**) Images are representative of >20 cells or nuclei per indicated mNG-EWSR1 reporter cell line.

Schematics in (**B**), (**C**), and (**E**) were generated using BioRender.

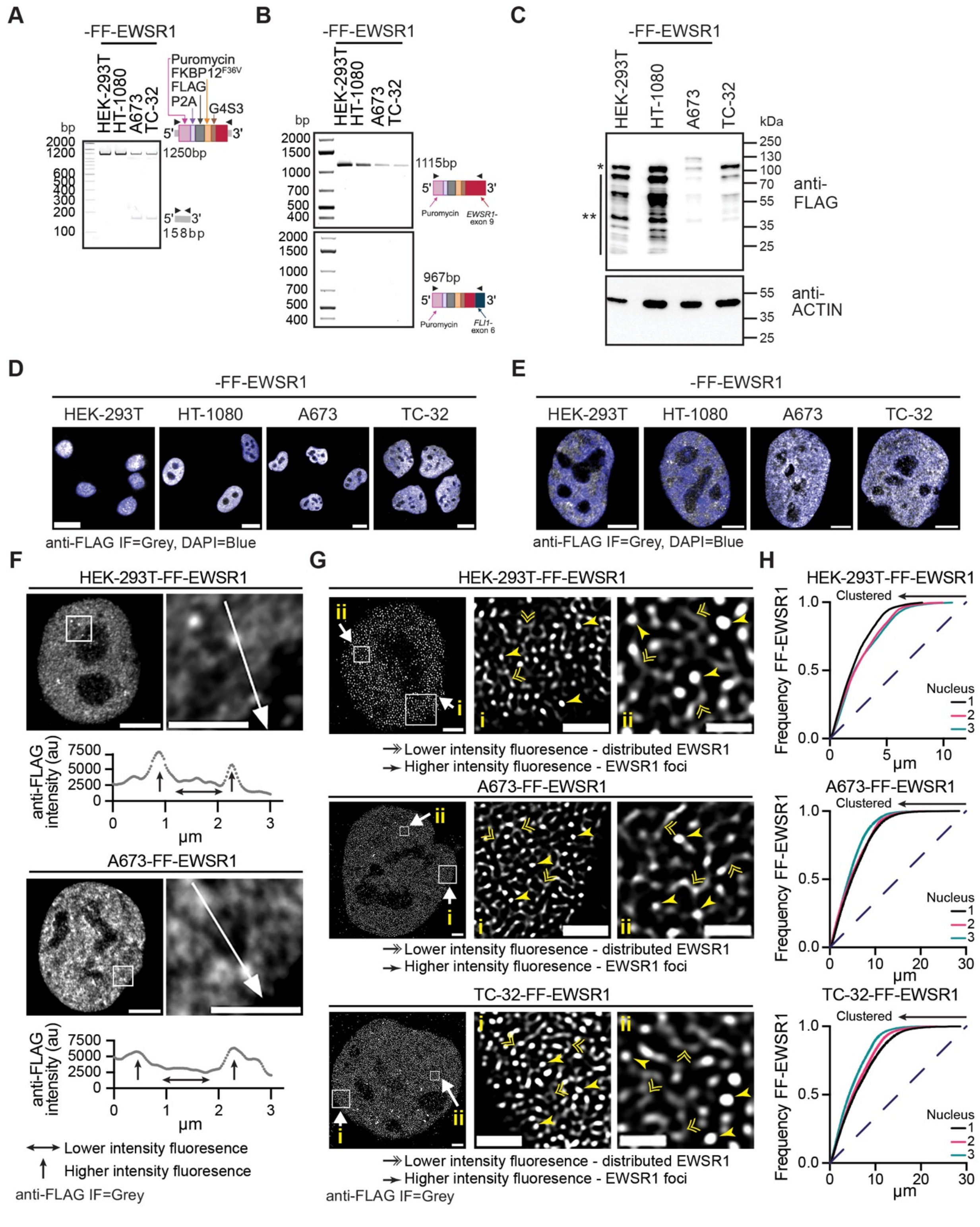

Figure S2: The generation of FLAG-FKBP12^{F36V}-EWSR1 (FF-EWSR1) reporter cell lines

(**A**) PCR-based analysis confirming targeted integration of the FF cassette into the endogenous *EWSR1* locus in the indicated reporter cell lines. PCR primers were designed to amplify across the donor cassette and the flanking 5' and 3' genomic regions encompassed by the *EWSR1* homology arms. This analysis demonstrates biallelic modification of *EWSR1* in HEK-293T and HT-1080 cells and monoallelic modification in EWS cell lines.

(**B**) RT-PCR analysis of FF-*EWSR1* transcripts using a common puromycin-specific forward primer and reverse *EWSR1*-exon 9 or *FLI1*-exon 6 primers. Modified *EWSR1* transcripts are detected, whereas modified *EWSR1::FLI1* transcripts are not.

(**C**) Immunoblot analysis showing an ~30 kDa molecular weight increase of FF-modified EWSR1. * and ** indicate modified major and minor EWSR1 isoforms, respectively.

(**D**) Confocal images of nuclei from the indicated FF-EWSR1 reporter cell lines showing anti-FLAG (grey) and DAPI (blue). Scale bar, 25 μm.

(**E**) SoRa super resolution confocal images of nuclei from the indicated FF-EWSR1 reporter cell lines showing anti-FLAG (grey) and DAPI (blue). Scale bar, 4 μm.

(**F**) SoRa super resolution confocal images showing nuclei (left) and expanded nuclear regions (right with corresponding line plots. anti-FLAG IF (grey) and line plots correspond to the white lines shown in the expanded regions (arrow direction indicates the x-axis orientation of each plot). Horizontal arrowed lines indicate lower anti-FLAG signal, whereas vertical arrowed lines indicate higher intensity signal. Scale bars, nucleus, 4 μm, expanded region, 2 μm.

(**G**) STED microscopy images of nuclei and expanded nuclear regions (i and ii) from the indicated FF-EWSR1 reporter cell lines showing anti-FLAG IF (grey). Double arrowheads indicate lower-intensity distributed FF-EWSR1 signal and single arrowheads indicate high intensity FF-EWSR1 foci. Scale bars, nucleus, 2 μm, expanded regions i, 1 μm, ii, 0.5 μm.

(**H**) Spatial cluster analysis of FF-EWSR1 pattern distribution in the indicated cell lines. Frequency plots show analysis of STED microscopy images of four nuclei. The dashed line indicates a random point pattern distribution; arrowed solid lines indicate deviations from a random point pattern distribution.

(**D**-**G**) Images are representative of > 20 cells or nuclei per indicated FF-EWSR1 reporter cell line.

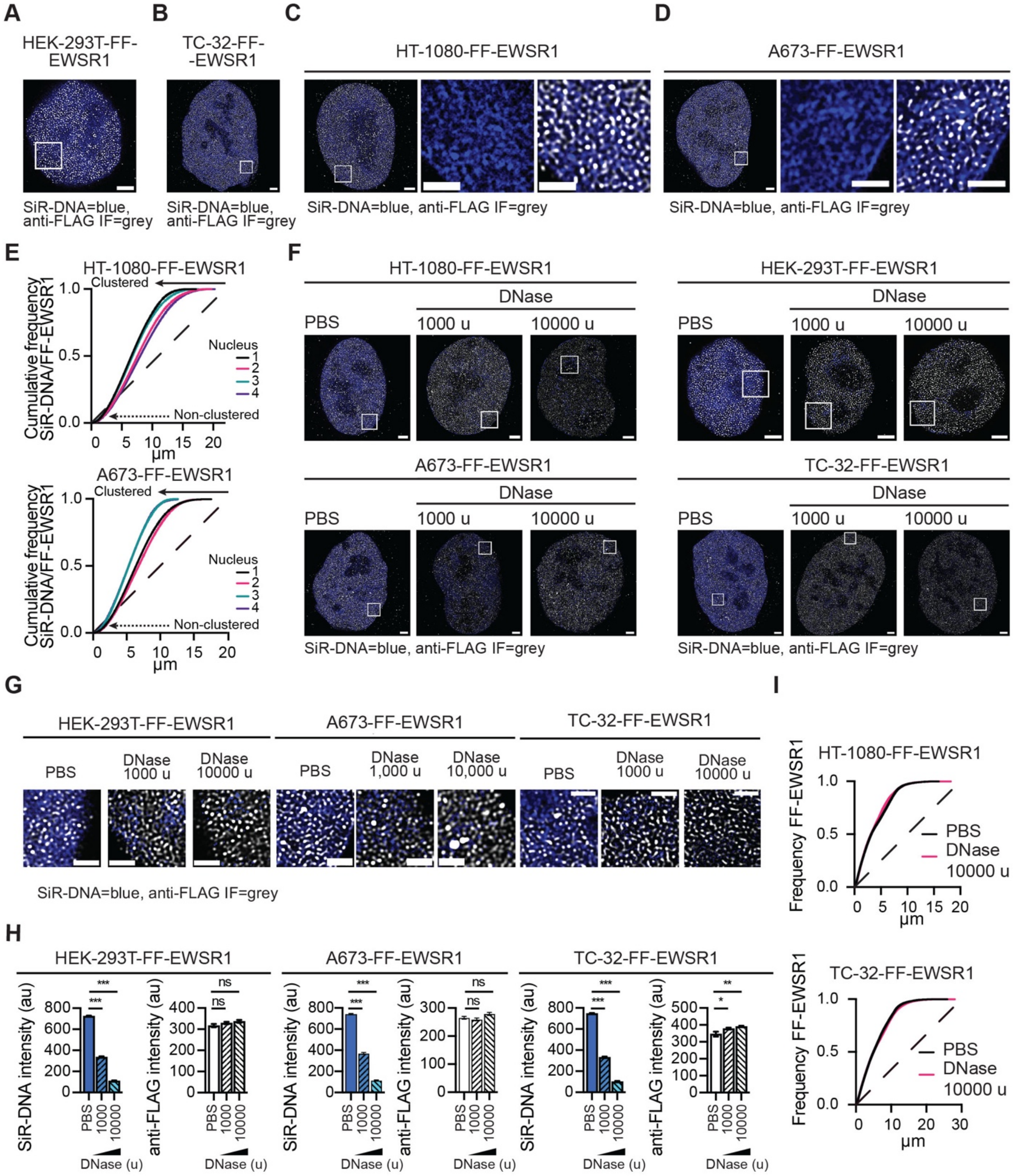

Figure S3: The nuclear organization of EWSR1 is DNA-independent

(**A, B**) STED microscopy images of nuclei from HEK-293T–FF–EWSR1 (**A**) and TC-32–FF–EWSR1 cells (**B**) with the boxes indicating the expanded regions shown in **Figures 2A** and **2B**, respectively. Merged images show the FLAG IF (grey) and SiR-DNA (blue). Scale bars, 2 μm.

(**C, D**) STED microscopy images of nuclei and indicated expanded regions from HT-1080–FF–EWSR1 (**C**) and A673–FF–EWSR1 (**D**) cells. Expanded regions show single-channel SiR-DNA (blue) and merged SiR-DNA (blue) with anti-FLAG IF (grey). Scale bars, nucleus, 2 μm, expanded region, 1 μm.

(**E**) Spatial cluster analysis of FF-EWSR1 IF pattern distribution relative to DNA, using SiR-DNA as the reference structure. Cumulative frequency plots show analysis of STED microscopy images of four representative nuclei per indicated reporter cell line.

(**F**) STED microscopy images of nuclei from the indicated FF-EWSR1 reporter cell lines treated with PBS, or DNase (1000 or 10000 u, 20 minutes (min)). Boxes indicate the expanded regions shown in **Figures 2D** or **Figure S3G**. Merged images show anti-FLAG IF (grey) and SiR-DNA (blue). Scale bar, 2 µm.

(**G**) Expanded regions from HEK-293T-FF-EWSR1, A673-FF-EWSR1, and TC-32-FF-EWSR1 cells treated with PBS or DNase (1000 or 10000 u, 20 min). Merged images show anti-FLAG IF (grey) and SiR-DNA (blue). Corresponding whole-nucleus images are shown in **Figure S3F**. Scale bar, 1 µm.

(**H**) Quantification of SiR-DNA and anti-FLAG fluorescence intensities in the indicated FF-EWSR1 reporter cells following treatment with either PBS or DNase (1000 or 10000 u, 20 min).

(**I**) Spatial cluster analysis of FF-EWSR1 IF pattern distribution. Plots show analysis of STED microscopy images of representative nuclei from HT-1080-FF-EWSR1 and TC-32-FF-EWSR1 cells treated with PBS or DNase (10000 u).

(**A**-**D, F, G**) Images are representative of >20 nuclei per indicated FF-EWSR1 reporter cell line.

(**E**, **I**) The dashed line indicates a random point pattern distribution; arrowed solid line indicates deviations from a random point pattern distribution; arrowed dotted lines indicate non-clustered signals (**E**).

(**H**) Data are shown as mean ± SEM from 20 nuclei per treatment per reporter cell line. Statistical significance was determined by one-way ANOVA, ⋆ $p<0.05$, ⋆⋆ $p<0.01$, ⋆⋆⋆ $p<0.001$, ns non-significant.

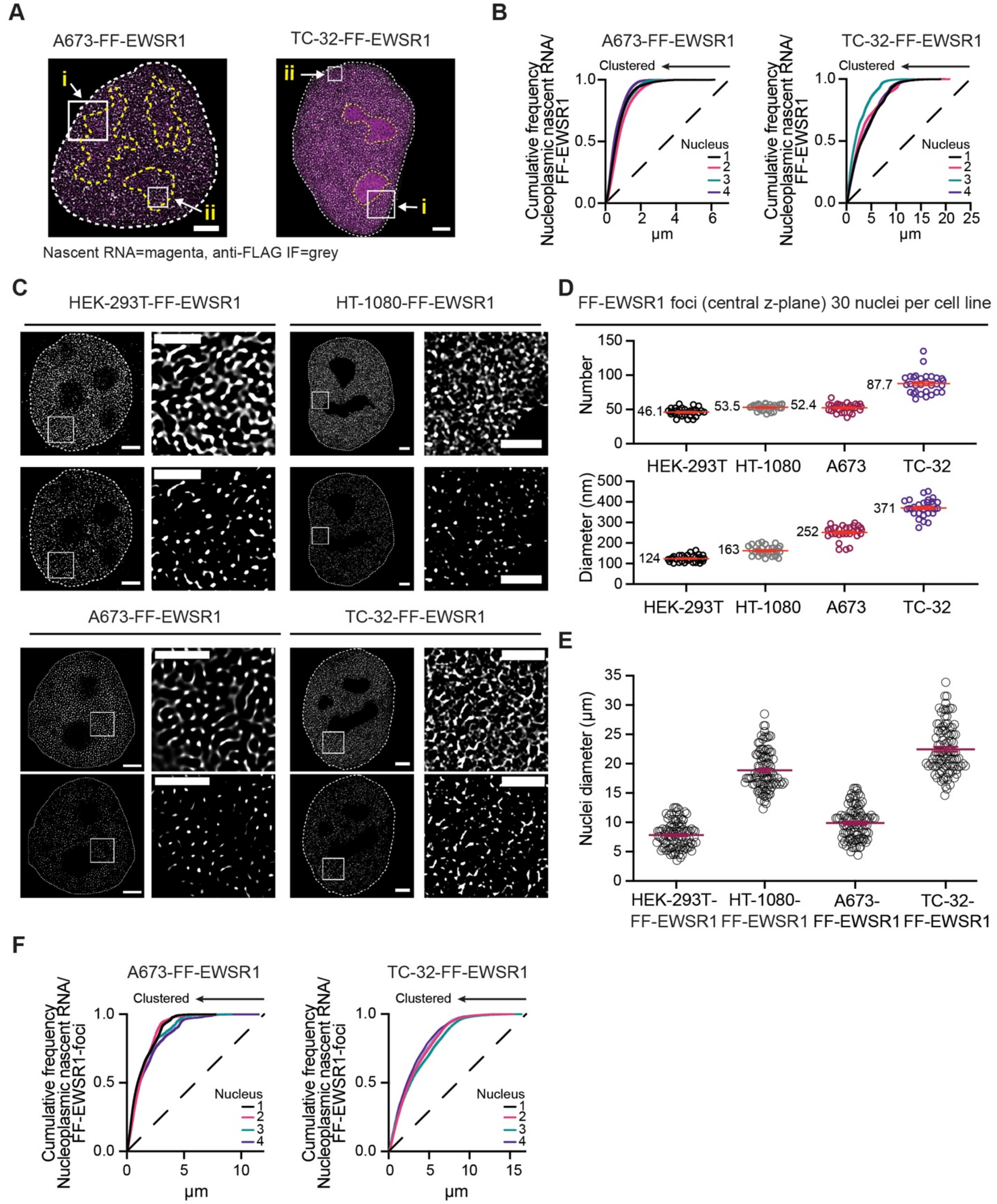

Figure S4: EWSR1 and newly synthesized RNA form a ribonucleoprotein network

(**A**) STED microscopy images of nuclei from A673-FF-EWSR1 and TC-32-FF-EWSR1 cells. Merged images show anti-FLAG IF (grey) and nascent RNA labeling (magenta). The white boxes indicate the expanded regions shown in **Figure 2G**. Scale bars, 2 µm.

(**B**) Spatial cluster analysis of FF-EWSR1 IF pattern distribution relative to newly synthesized RNA in the indicated FF-EWSR1 reporter cell lines using EU-labeled RNA as the reference structure.

Cumulative frequency plots show analysis of STED microscopy images of four representative nuclei per indicated reporter cell line.

(**C**) STED microscopy images of nuclei and expanded nuclear regions from the indicated FF-EWSR1 reporter cell lines. Upper panels show anti-FLAG IF signals (grey) above background and lower panels show FF–EWSR1 foci (grey) defined by ≥5× fluorescence intensity relative to background. Scale bars, nucleus, 2 μm, expanded region, 1 μm.

(**D**) Quantification of the number and the diameter (nm) of FF-EWSR1 foci at the central z-plane of the nuclei from the indicated reporter cell lines. Foci defined as anti-FLAG IF signal ≥5x above background. n=30 nuclei per indicated reporter cell line. Numbers indicate the mean for each parameter in each reporter cell line

(**E**) Quantification of the nuclear diameter (nm) in the indicated FF-EWSR1 reporter cell lines. n=100 nuclei per cell line.

(**F**) Spatial cluster analysis of high intensity (≥5x signal) FF-EWSR1 foci pattern distribution relative to newly synthesized RNA in the indicated FF-EWSR1 reporter cell lines using EU-labeled RNA as the reference structure. Cumulative frequency plots show analysis of STED microscopy images of four representative nuclei per indicated reporter cell line.

(**A**, **C**) Images are representative of >20 nuclei per indicated FF-EWSR1 reporter cell line.

(**B**, **F**) The dashed lines indicate a random point pattern distribution; arrowed solid lines indicate deviations from a random point pattern distribution.

(**D**) n=30 nuclei per indicated reporter cell line. Data show as individual data points and mean ±SEM (red lines). Values indicate the mean for each parameter in each cell line.

(**E**) n=100 nuclei per reporter cell line. Data show as individual data points and mean ±SEM (red lines).

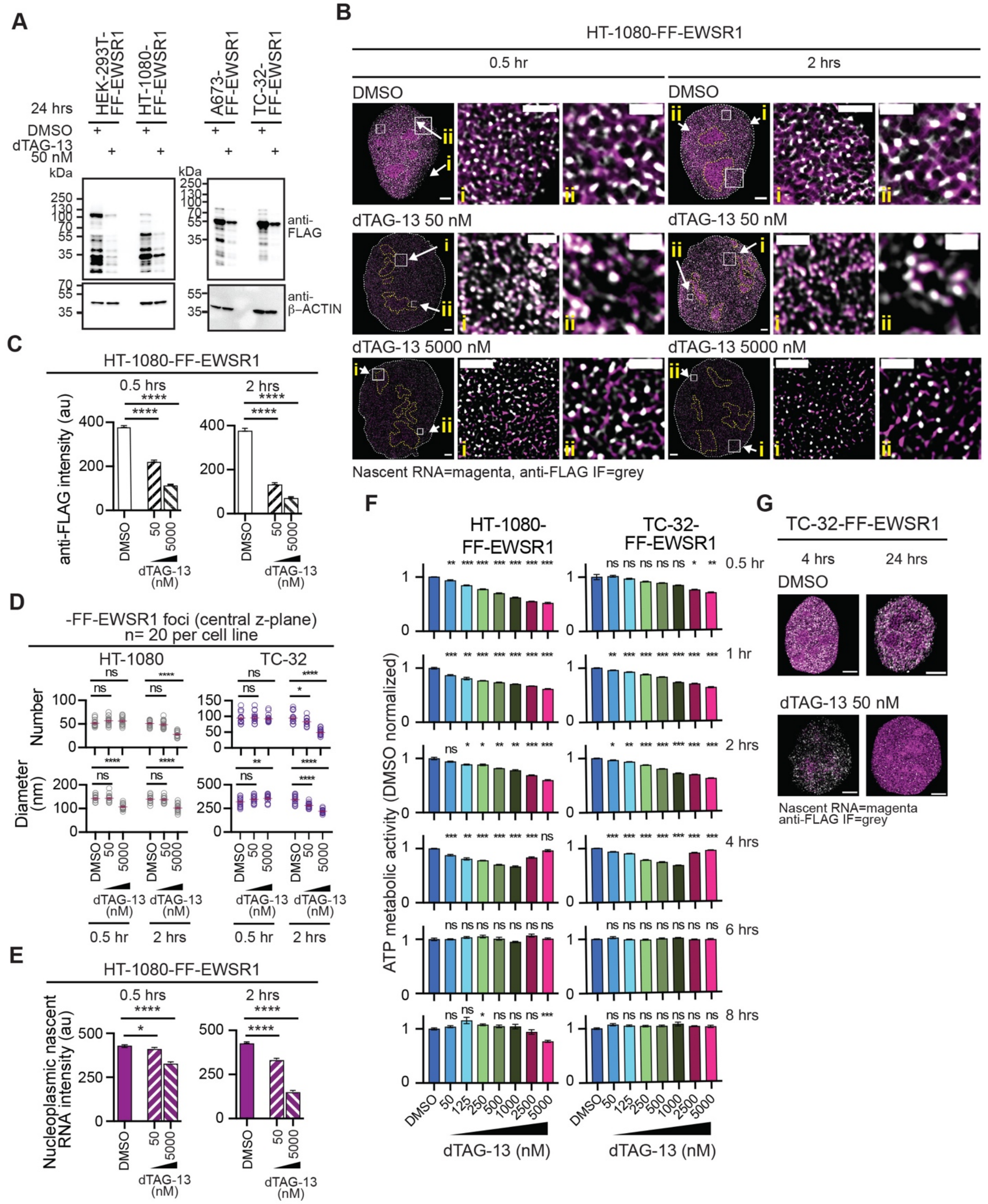

Figure S5: The degradation of EWSR1 results in a transient decrease in nascent RNA and cell viability.

(**A**) Immunoblot analysis of whole cell lysates from the indicated FF-EWSR1 reporter cell lines treated with DMSO or dTAG-13 (50 nM) for 24 hours (hrs) and probed with antibodies against the indicated proteins.

(**B**) STED microscopy images of nuclei and two expanded regions (i and ii) from HT-1080-FF-EWSR1 cells treated with DMSO (upper panels), or dTAG-13 (50 nM, middle panels, or 5000 nM, lower panels) for 0.5 or 2 hrs. Merged images show anti-FLAG IF (grey) and nascent RNA (magenta). Scale bars, nucleus 4 μm, expanded regions i, 1 μm, ii, 0.5 μm.

(**C**) Quantification of anti-FLAG IF intensity in HT-1080-FF-EWSR1 cells following treatment with DMSO or dTAG-13 (50 or 5000 nM) for 0.5 (left) or 2 (right) hrs.

(**D**) Quantification of the number and the diameter (nm) of FF-EWSR1 foci measured at the central z-plane of the indicated reporter cell lines following treatment with DMSO or dTAG-13 (50 or 5000 nM) for 0.5 or 2 hrs. Foci were defined as anti-FLAG fluorescence intensity ≥5x above background.

(**E**) Quantification of nascent RNA fluorescence intensity in HT-1080-FF-EWSR1 cells following treatment with DMSO or dTAG-13 (50 or 5000 nM) for 0.5 (left) or 2 (right) hrs.

(**F**) Cell viability of HT-1080-FF-EWSR1 and TC-32-FF-EWSR1 reporter cell lines measured 0.5 – 8 hrs after addition of DMSO or increasing concentration of dTAG-13 (50 -5000 nM). Viability values are normalized to the mean of DMSO-treated cells at each time point.

(**G**) SoRa images of nuclei from TC-32-FF- EWSR1 reporter cells 4 or 24 hrs after treatment with DMSO or dTAG-13 (50 nM). Scale bars, 4 μm. Quantification of anti-FLAG IF and nascent RNA fluorescence intensities is shown in **Figure 3E**.

(**B, G**) Images are representative of >20 nuclei per indicated FF-EWSR1 reporter cell line.

(**C, E**, **F**) Data shown as mean ± SEM. (**D**) Individual data points (circles) and the mean ± SEM (lines) (**C, E**) n=15 nuclei per reporter cell line per condition. (**D**) n=20 nuclei per reporter cell line. (**F**) n=6 biological replicates per reporter cell line per treatment per time point.

(**C-F**) Statistical significance was determined using one-way ANOVA. (**C -F**) * $p<0.05$, ** $p<0.01$, *** $p<0.00$, **** $p<0.0001$, ns non-significant.

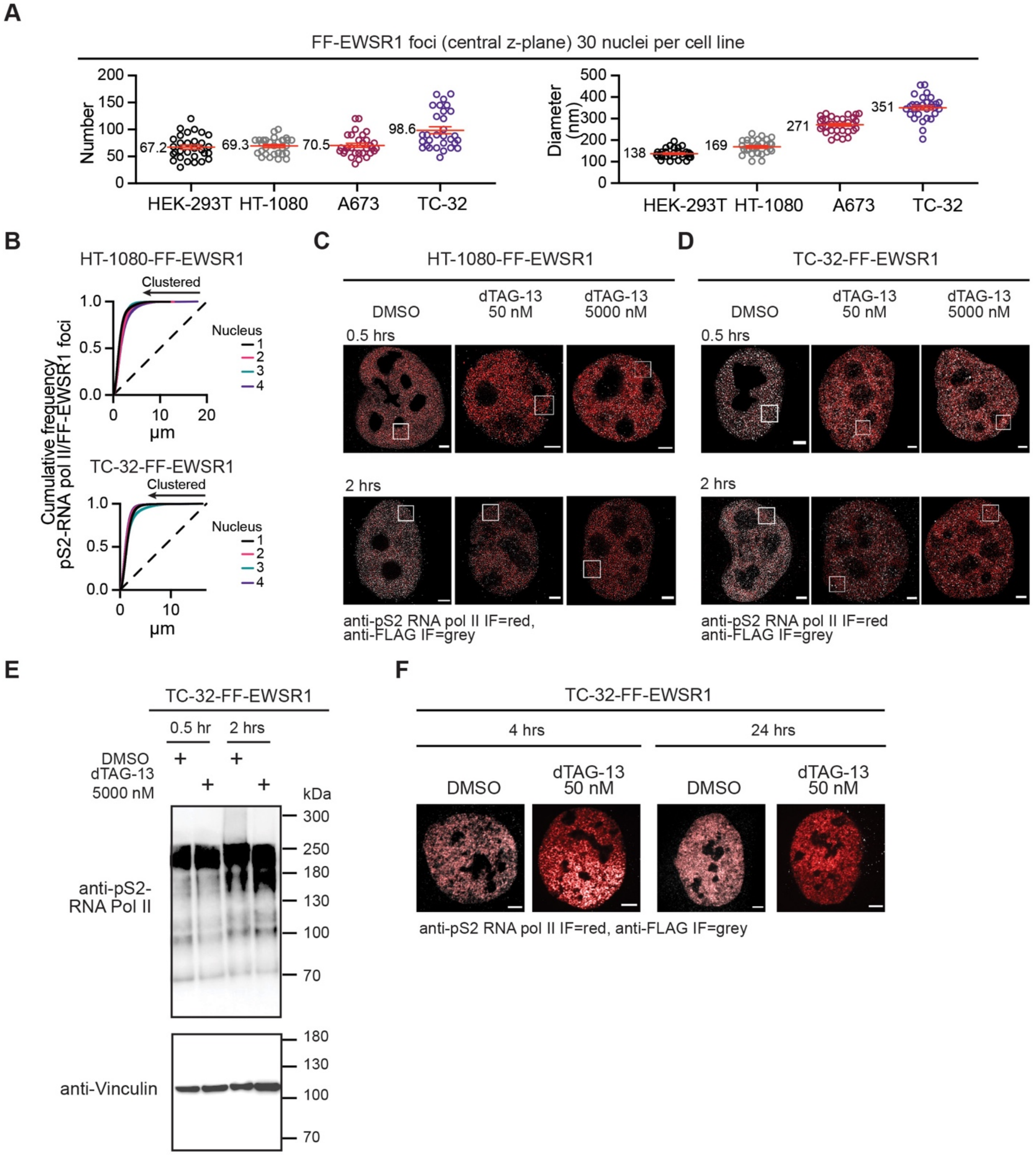

Figure S6: EWSR1 foci colocalize with actively elongating RNA polymerase II, but depletion of EWSR1 does not measurably alter this marker of transcriptional activity

(**A**) Quantification of the number and diameter (nm) of FF-EWSR1 foci at the central z-plane of the indicted cells. Foci defined as anti-FLAG fluorescence intensity ≥5x than background.

(**B**) Spatial cluster analysis of FF–EWSR1 foci (≥5x above background) pattern distribution as a function of distance relative to pS2-RNA pol II IF in the indicated FF–EWSR1 reporter cell lines. Cumulative frequency plots were generated from STED microscopy images of four nuclei per reporter cell line.

(**C, D**) STED microscopy images of nuclei from HT-1080-FF-EWSR1 cells (**C**) and TC-32-FF-EWSR1 cells (**D**) treated with DMSO or dTAG-13 (50 or 5000 nM) for the indicated times. Merged images show anti-FLAG (grey) and pS2-RNA pol II IF (red). Boxes indicate the expanded regions shown in **Figure 4F**. Scale bar, 2 µm.

(**E**) Immunoblot analysis of whole cell lysates from TC-32-FF- EWSR1 cells treated with either DMSO or dTAG-13 (5000 nM) for the indicated times and probed using the antibodies against the indicated proteins.

(**F**) SoRa images of the nuclei of the indicated FF- EWSR1 reporter cells 4 or 24 hrs post-addition of DMSO or dTAG-13 (50 nM). Merged images show anti-FLAG (grey) and pS2-RNA pol II IF (red). Scale bar, 4 µm.

(**A**) Data shown as individual data points and mean ± SEM (red lines), mean value indicated. n=30 nuclei per reporter cell line.

(**B**) The dashed line represents a random point pattern distribution. Arrowed solid lines indicate deviations from a random point pattern distribution.

(**C**, **D, F**) Images are representative of >20 nuclei per indicated FF-EWSR1 reporter cell line.

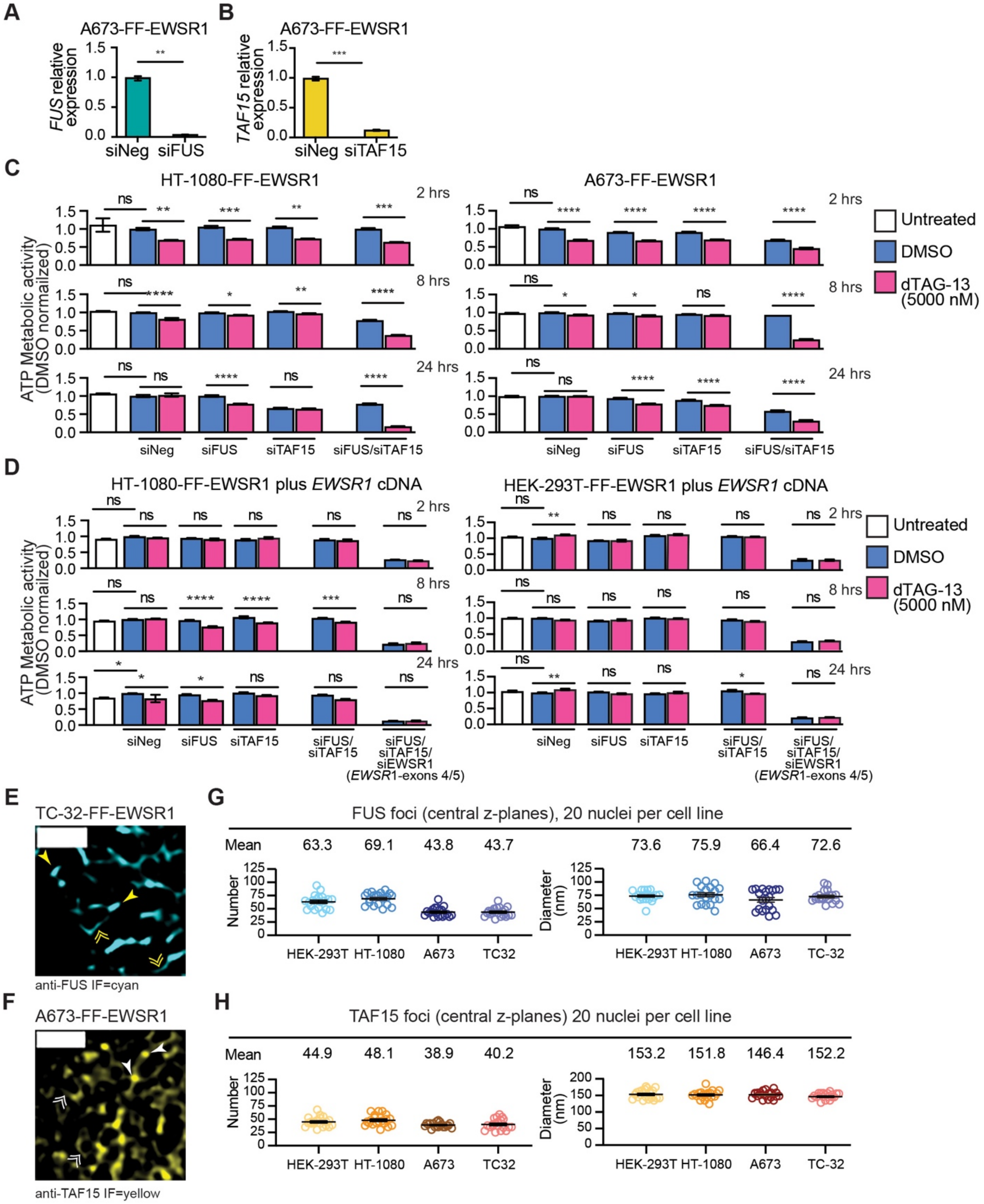

Figure S7: FUS and TAF15 functionally compensate for EWSR1 loss to restore cellular metabolic activity

(**A, B**) qRT-PCR analysis of *FUS* (**A**) and *TAF15* (**B**) expression 48 hours post-transfection of A673-FF-EWSR1 cells with the indicated siRNAs. *FUS* or TAF15 expression normalized to the expression of the housekeeping gene *RPL27* and shown relative to siNeg-transfected cells.

(C) The ATP metabolic activity of HT-1080-FF-EWSR1 and A673-FF-EWSR1 reporter cells following siRNA-mediated depletion of FUS and/or TAF15 (48 hrs) and dTAG-13-mediated degradation of EWSR1 (2, 8, or 24 hrs). Data are normalized to the mean of the siNeg-transfected, DMSO-treated cells.

(**D**) The ATP metabolic activity of HT-1080-FF-EWSR1 and HEK-293T-FF-EWSR1 reporter cells stably expressing an *EWSR1* cDNA following siRNA-mediated depletion of FUS and/or TAF15 (48 hrs) and dTAG-13-mediated degradation of EWSR1 (2, 8, or 24 hrs). Data are normalized to the mean of the siNeg-transfected, DMSO-treated cells.

(**E**) STED microscopy images of an expanded nuclear region from a TC-32-FF-EWSR1 showing FUS IF (cyan). The double arrowheads indicate lower intensity fluorescence or distributed FUS, and the single arrowheads indicate higher intensity fluorescence or FUS foci. Scale bars, nucleus, 2 μm, expanded region, 1 μm.

(**F**) STED microscopy images of an expanded nuclear region from an A673-FF-EWSR1 showing TAF15 IF (yellow). The double arrowheads indicate lower intensity fluorescence or distributed TAF15, and the single arrowheads indicate higher intensity fluorescence or TAF15 foci. Scale bars, nucleus, 2 μm, expanded region, 1 μm.

(**G, H**) Quantification of FUS (**G**) and TAF15 (**H**) foci number and diameter (≥5× background).

(**A, B**) Data shown as mean ± SEM, n=3 per transfection.

(**C, D**) Data shown as the mean ± SEM, n=6 per treatment.

(**E, F**) Images are representative of >20 nuclei per indicated EWSR1 reporter cell line.

(**G, H**) Data are shown as individual values and mean ± SEM, n=20 nuclei per condition per reporter cell line.

(**A, B**) Statistical significance was determined using Welch's t-test. (**C, D**) Statistical significance was determined using one-way ANOVA.⋆ p<0.05, ⋆⋆ p<0.01, ⋆⋆⋆ p<0.001, ⋆⋆⋆⋆ p<0.0001, ns non-significant.

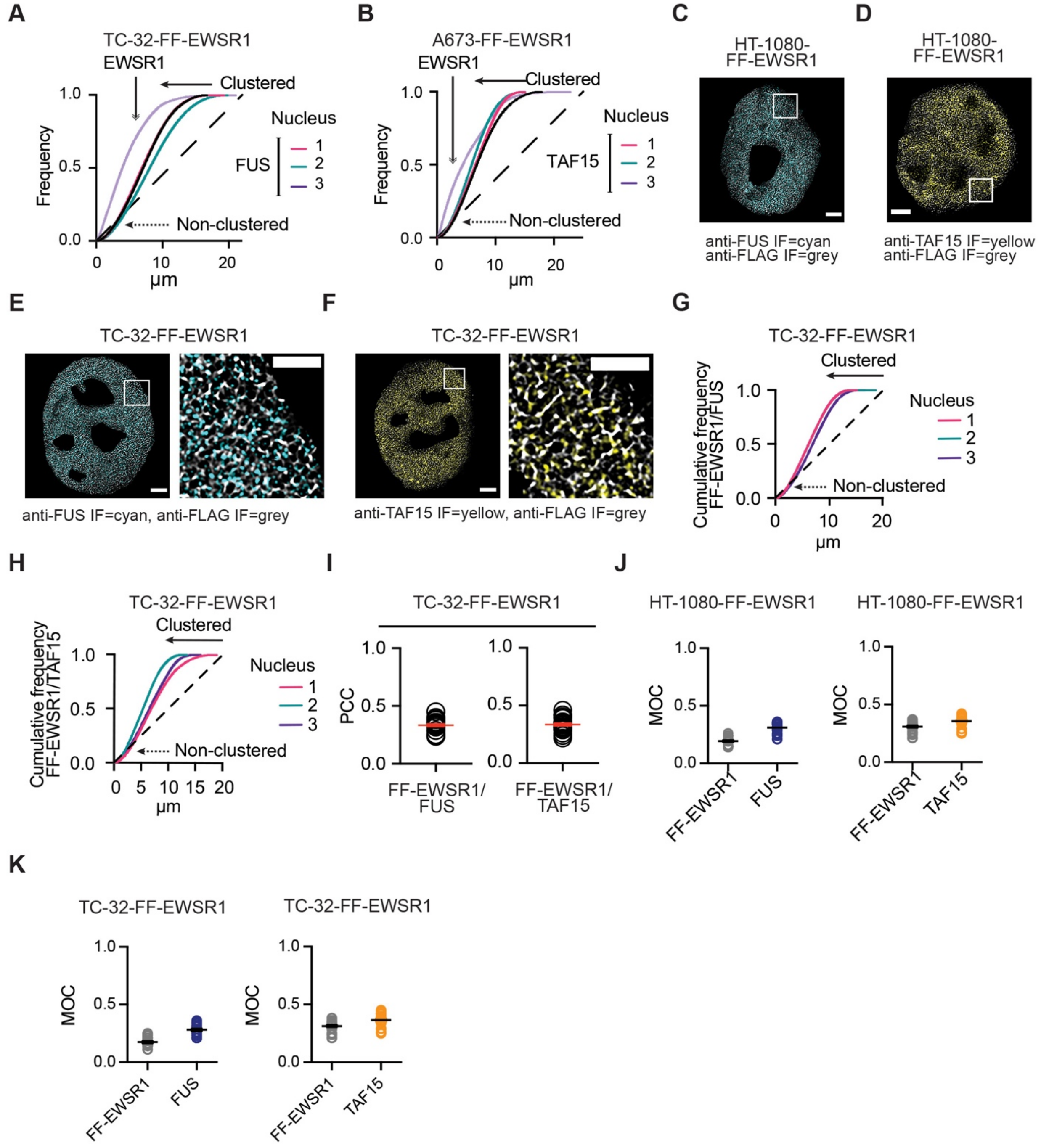

Figure S8: EWSR1 exhibits minimal spatial colocalization with FUS or TAF15

(**A, B**) Spatial cluster analysis of FUS IF (**A**) and TAF15 IF (**B**) pattern distributions across distance for TC-32-FF-EWSR1 cells (**A**) and A673-FF-EWSR1 cells (**B**). Frequency plots were generated from STED microscopy images of three nuclei per reporter cell line. A frequency plot of FF-EWSR1 in each report cell line is shown for comparison (light purple).

(**C, D**) STED microscopy images of nuclei from HT-1080-FF-EWSR1 cells. Merged images show anti-FUS (cyan) and anti-FLAG IF (grey)(**C**) or anti-TAF15 (yellow) and anti-FLAG IF (grey) (**D**). Boxes indicate the expanded regions shown in **Figures 5H, I**. Scale bar, 2 μm.

(**E, F**) STED microscopy merged images of nuclei and enlarged nuclear regions from TC-32-FF-EWSR1 cells showing anti-FUS (cyan) and anti-FLAG IF (grey) (**E**) or anti-TAF15 (yellow) and anti-FLAG IF (grey) **(F)**. Scale bars, nucleus, 2 μm, expanded region, 1 μm.

(**G, H**) Spatial cluster analysis of FUS IF (**G**) and TAF15 IF (**H**) pattern distributions as a function of distance relative to FF-EWSR1 in TC-32-FF-EWSR1 cells. Cumulative frequency plots were generated from STED microscopy images of three nuclei per reporter cell line per analysis.

(**I**) Quantification of FF–EWSR1 colocalization with FUS or TAF15 in TC-32-FF-EWSR1 cells by PCC analysis.

(**J**) Quantification of FF–EWSR1 colocalization with FUS or TAF15 in HT-1080-FF-EWSR1 cells by MOC analysis.

(**K**) Quantification of FF–EWSR1 colocalization with FUS or TAF15 in TC-32-FF-EWSR1 cells by MOC analysis.

(**A**, **B**, **G**, **H**) The dashed line represents a random point pattern distribution; arrowed solid lines indicate deviations from a random point pattern distribution; arrowed dotted lines indicate non-clustered signals.

(**C**-**F**) Images are representative of >20 nuclei per indicated EWSR1 reporter cell line.

(**I-K**) Data are shown as individual values with mean ± SEM indicated (**I**, red lines, **J**, **K**, black lines), n=20 nuclei per condition per analysis.

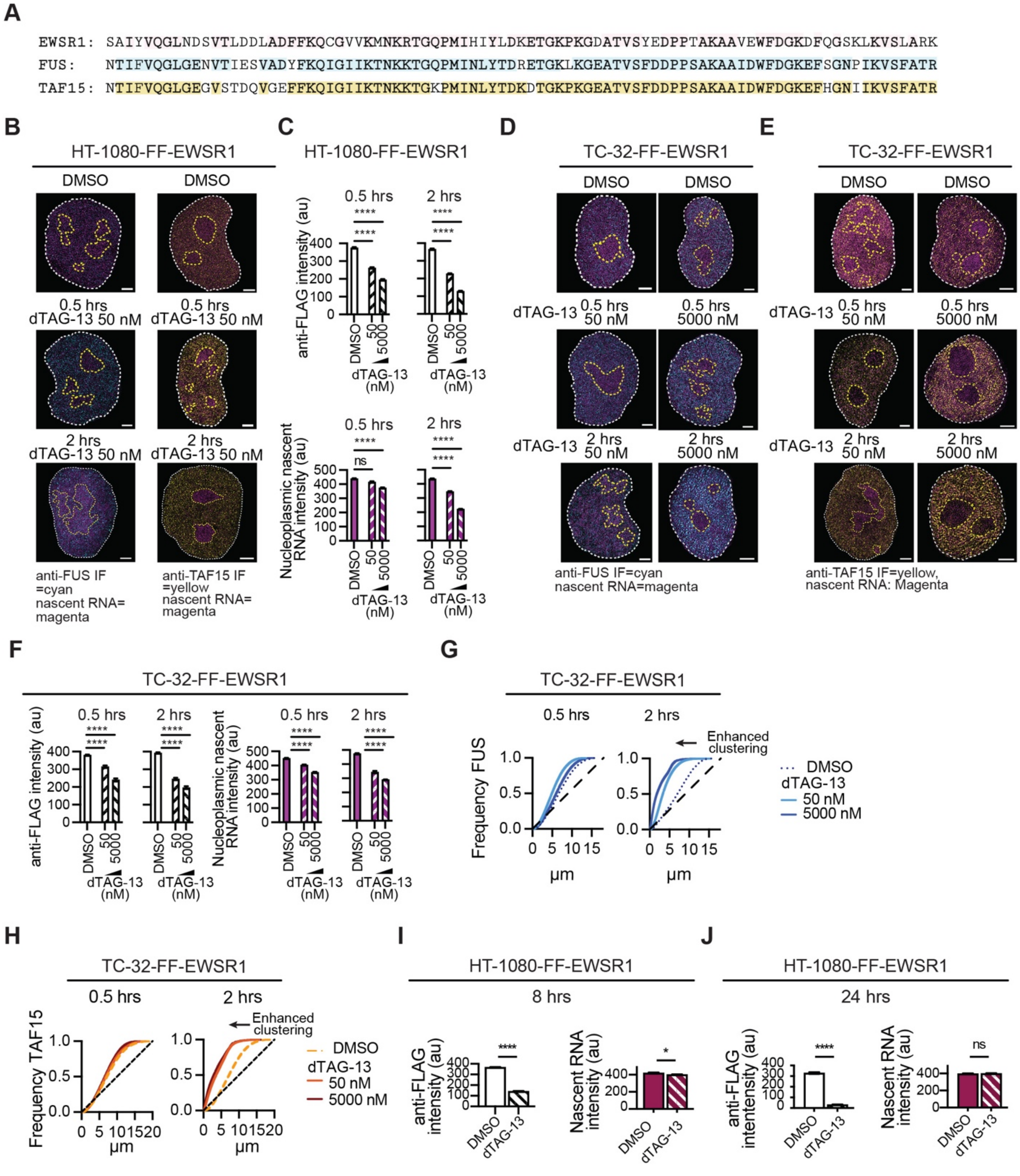

Figure S9: FUS and TAF15 reorganize following EWSR1 degradation

(**A**) The amino acid sequences of the FET protein RRM domains (extracted from UniProt).

(**B**) STED microscopy image of nuclei from HT-1080-FF-EWSR1 cells treated with DMSO or dTAG-13 (50 nM) for 0.5 or 2 hrs showing anti-FUS (cyan) (left panels) or anti-TAF15 (yellow)(right panels) Scale bar, 2 μm.

(**C**) Quantification of anti-FLAG IF intensity (upper) and nucleoplasmic nascent RNA intensity (lower) in HT-1080-FF-EWSR1 cells treated with DMSO or dTAG-13 (50 or 5000 nM) for 0.5 or 2 hrs.

(**D, E**) STED microscopy images of nuclei from TC-32-FF-EWSR1 cells treated with DMSO or dTAG-13 (50 or 5000 nM) for 0.5 or 2 hrs showing anti-FUS (cyan)(**D**) and anti-TAF15 (yellow)(**E**). Scale bar, 2 μm.

(**F**) Quantification of anti-FLAG IF intensity (left) and nucleoplasmic nascent RNA intensity (right) in TC-32-FF-EWSR1 cells treated with DMSO or dTAG-13 (50 or 5000 nM) for 0.5 or 2 hrs.

(**G, H**) Spatial cluster analysis of FUS (**G**) and TAF15 (**H**) pattern distributions. Frequency plots show analysis of STED microscopy images of a representative nucleus from TC-32-FF-EWSR1 cells treated with DMSO or dTAG-13 (5000 nM) for 0.5 or 2 hrs. The dashed line indicates a random point pattern distribution and arrowed solid lines indicate deviations a random point pattern distribution.

(**I, J**) Quantification of anti-FLAG IF intensity (left) and nucleoplasmic nascent RNA intensity (right) in HT-1080-FF-EWSR1 cells treated with DMSO or dTAG-13 (5000 nM) for 8 hrs (**I**) or 24 hrs (**J**).

(**A**, **D, E**) Images are representative of >20 nuclei per indicated FF-EWSR1 reporter cell line.

(**C, F, I, J**) Data are presented as mean ± SEM of 20 (**C,F**) or 15 (**I,J**) nuclei per treatment. Statistical significance was determined using one-way ANOVA ⋆ $p<0.05$, ⋆⋆⋆⋆ $p<0.0001$, ns non-significant.

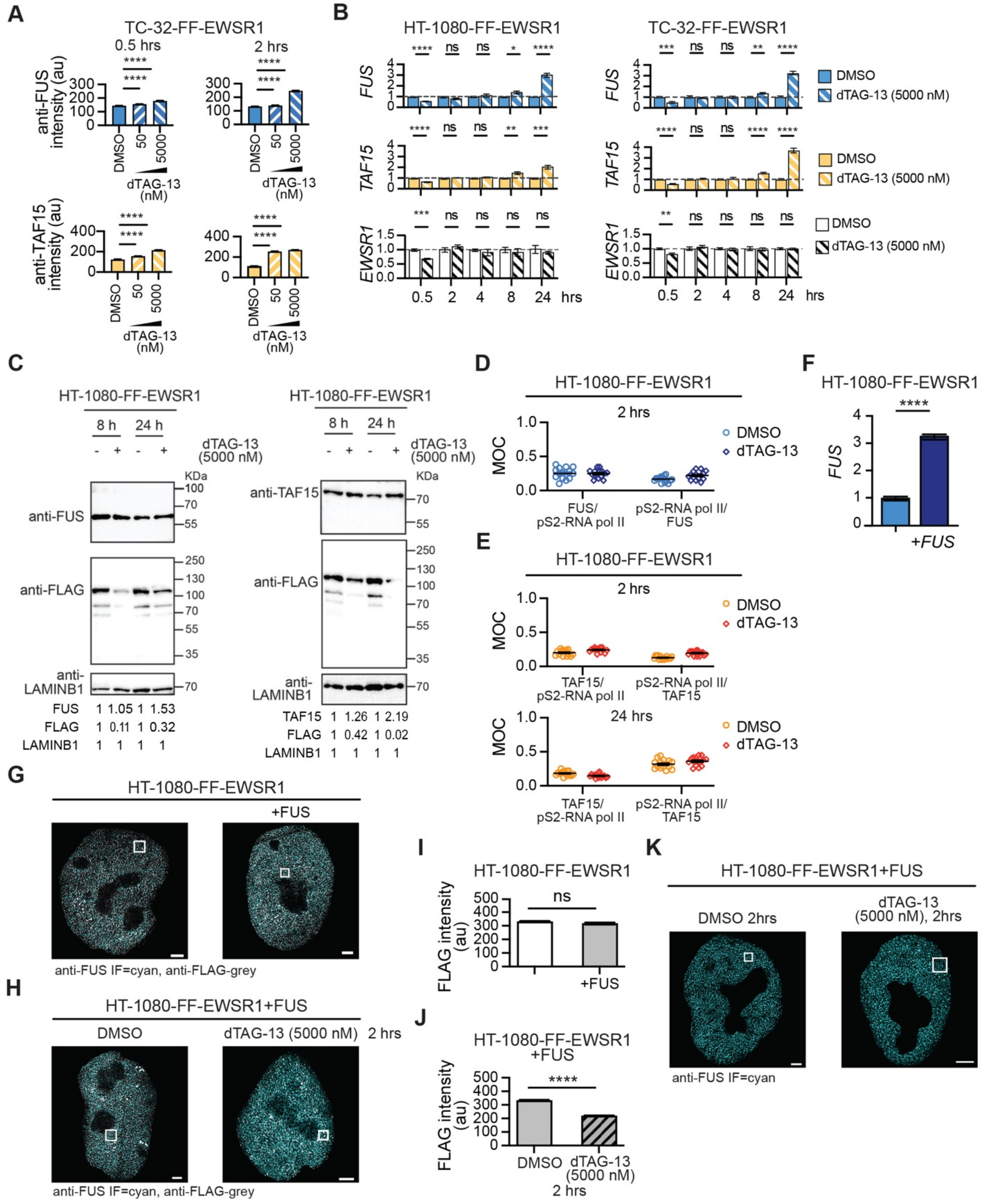

Figure S10: The nuclear reorganization of FUS requires loss of EWSR1

(**A**) Quantification of anti-FUS IF intensity (upper) and anti-TAF15 IF intensity (lower) in TC-32-FF-EWSR1 cells treated with DMSO or dTAG-13 (as indicated) for the times indicated.

(**B**) The expression of the indicated genes (*RPL27* normalized) following the addition of dTAG-13 (5000 nM) to HT-1080-FF-EWSR1 (left) or TC-32-FF-EWSR1 (right) cells at the indicated times. Data is shown relative to DMSO-treated cells.

(**C**) Immunoblot analysis of nuclear fractions lysates prepared from HT-1080-FF- EWSR1 reporter cell line treated with either DMSO or dTAG-13 (5000 nM) for 8 or 24 hrs and probed using the antibodies against the indicated proteins.

(**D**) Quantification of FUS colocalization with pS2-RNA pol II in HT-1080-FF-EWSR1 cells by MOC.

(**E**) Quantification of TAF15 colocalization with pS2-RNA pol II in TC-32-FF-EWSR1 cells by MOC.

(**F**) The expression of *FUS* (*RPL27* normalized) in HT-1080-FF-EWSR1 cells and HT-1080-FF-EWSR1 cells stably expressing *FUS*.

(**G, H**) STED microscopy images single channel (anti-FUS, cyan) and merged (anti-FUS, cyan; anti-FLAG, grey) of nuclei from HT-1080-FF-EWSR1 and HT-1080-FF-EWSR1+FUS cells (**G**) and HT-1080-FF-EWSR1+FUS cells treated with DMSO or dTAG-13 (5000 nM) (**H**).

(**I, J**) Quantification of anti-FLAG IF intensity in HT-1080-FF-EWSR1 and HT-1080-FF-EWSR1+FUS cells (**I**) and HT-1080-FF-EWSR1+FUS cells treated with DMSO or dTAG-13 (5000 nM) (**J**).

(**K**) STED microscopy images (anti-FUS IF (cyan)) of nuclei from HT-1080-FF-EWSR1+FUS cells treated with either DMSO or dTAG-13 (5000 nM) for 2 hrs.

(**A**) Data are presented as mean ± SEM of 20 nuclei per treatment.

(**B**) Data are presented as mean ± SEM of 6 replicates per treatment per time point. (**C**) Data are presented as mean ± SEM of 3 replicates. (**I, J**) Data are presented as mean ± SEM of 15 nuclei per treatment. Statistical significance was determined using one-way ANOVA. ⋆ $p<0.05$, ⋆⋆ $p<0.01$, ⋆⋆⋆ $p<0.001$, ⋆⋆⋆⋆ $p<0.0001$, ns non-significant.

(**G, H, K**) Images are representative of >15 nuclei per HT-1080-FF-EWSR1 or HT-1080 FF-EWSR1+FUS reporter cell line.

References

1. Grohar, P.J., Kim, S., Rangel Rivera, G.O., Sen, N., Haddock, S., Harlow, M.L., Maloney, N.K., Zhu, J., O'Neill, M., Jones, T.L., et al. (2016). Functional Genomic Screening Reveals Splicing of the *EWS-FLI1* Fusion Transcript as a Vulnerability in Ewing Sarcoma. Cell Rep *14*, 598-610. 10.1016/j.celrep.2015.12.063.
2. Ebegboni, V.J., Jones, T.L., Brownmiller, T., Zhao, P.X., Pehrsson, E.C., Sundara Rajan, S., and Caplen, N.J. (2024). ETS1, a Target Gene of the EWSR1::FLI1 Fusion Oncoprotein, Regulates the Expression of the Focal Adhesion Protein TENSIN3. Mol Cancer Res *22*, 625-641. 10.1158/1541-7786.MCR-23-1090.
3. Sundara Rajan, S., Ebegboni, V.J., Pichling, P., Ludwig, K.R., Jones, T.L., Chari, R., Tran, A., Kruhlak, M.J., Loncarek, J., and Caplen, N.J. (2024). Endogenous EWSR1 Exists in Two Visual Modalities That Reflect Its Associations with Nucleic Acids and Concentration at Sites of Active Transcription. Mol Cell Biol *44*, 103-122. 10.1080/10985549.2024.2315425.
